# *SIMAGE*: *si*mulation of DNA-*m*icro*a*rray *g*ene *e*xpression data

**DOI:** 10.1186/1471-2105-7-205

**Published:** 2006-04-13

**Authors:** Casper J Albers, Ritsert C Jansen, Jan Kok, Oscar P Kuipers, Sacha AFT van Hijum

**Affiliations:** 1 Groningen Bioinformatics Centre, University of Groningen, Groningen Biomolecular Sciences and Biotechnology Institute, PO Box 14, 9750 AA Haren, The Netherlands; 2 Department of Molecular Genetics, University of Groningen, Groningen Biomolecular Sciences and Biotechnology Institute, PO Box 14, 9750 AA Haren, The Netherlands; 3 Department of Statistics, The Open University, Walton Hall, Milton Keynes MK7 6AA, UK

## Abstract

**Background:**

Simulation of DNA-microarray data serves at least three purposes: (i) optimizing the design of an intended DNA microarray experiment, (ii) comparing existing pre-processing and processing methods for best analysis of a given DNA microarray experiment, (iii) educating students, lab-workers and other researchers by making them aware of the many factors influencing DNA microarray experiments.

**Results:**

Our model has multiple layers of factors influencing the experiment. The relative influence of such factors can differ significantly between labs, experiments within labs, etc. Therefore, we have added a module to roughly estimate their parameters from a given data set. This guarantees that our simulated data mimics real data as closely as possible.

**Conclusion:**

We introduce a model for the simulation of dual-dye cDNA-microarray data closely resembling real data and coin the model and its software implementation "*SIMAGE*" which stands for simulation of microarray gene expression data. The software is freely accessible at: .

## Background

**Figure 1:**
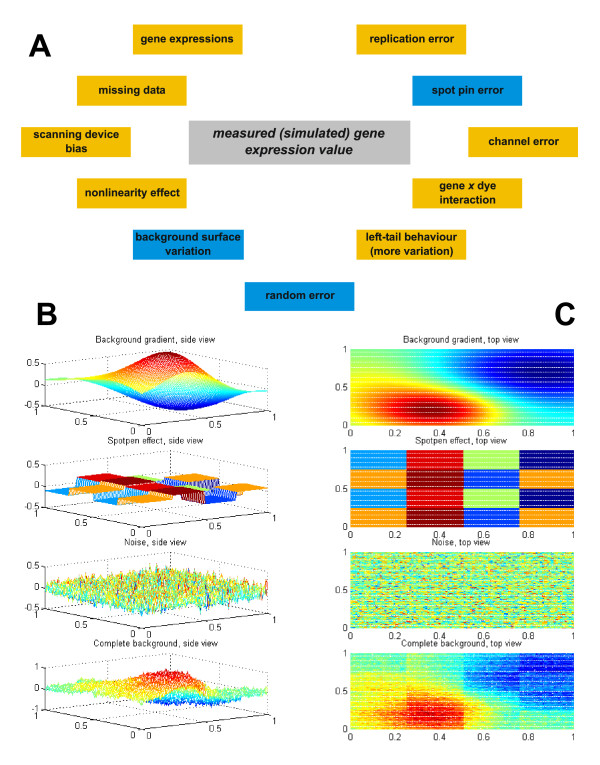
**Schematic overview of the *SIMAGE *model (A) and a few layers visualized (B and C). **The blue-marked boxes (A) indicate layers that are further visualized (B and C). A simulation of the entire 'non-biological' signal is shown in B and C. Top row, sum of the gradient effects and density effects; second row, spot pin effects; third row: Gaussian noise. The bottom row shows the sum of all the effects pictured in the top three rows. The signals are plotted three-dimensionally (left side view) and two-dimensionally (right side view).

No two laboratories produce the same expression data when performing seemingly identical DNA microarray experiments. This is simply due to the fact that experimental conditions and factors such as growth media composition, RNA sampling methodology and scanner calibration are never exactly identical [1]. Even within one and the same laboratory differences in the outcome of experiments executed by different laborants can be observed [2,3]. These and many other factors lead to sometimes unexpected gene expression variations that can occur at several levels. Figure 1 shows a schematic and simplified overview of those levels in dual-color DNA microarray data (top) as well as the effect of some of these levels on the composition of the expression signals (bottom).

It is obvious that knowledge about the properties that gene expression signals hold as shown in Figure 1 is very important to any researcher. This knowledge can be used to design an optimal new DNA microarray experiment and to carry out the best analysis of that generated data set. To this end, real and simulated data has been used for a long time in data analysis workshops. And in case it is not (yet) obvious for a novice in the field, the visualization as shown in Figure 1 can be of educational value. In all three cases – design, analysis and education – the availability of simulation software would be very helpful. It is of eminent importance that the simulated data resembles experimental data as much as possible [4] and, therefore, there is a need for software that can (roughly) estimate levels of variation.

Various software packages for simulating dual-color DNA microarray data have been described in the literature (Table 1). Some of these packages aim at validating image analysis and spot quantification and simulate TIF-images of the visualized spots. Other software focuses on gene networks. In this paper, we present new software that fits in between these two categories: we simulate the gene expression data as it is tabulated after image analysis. In addition, we provide software that can be used to roughly estimate the levels of variation in real data. These computer programs, named *SIMAGE*, are freely available via a web-resource.

## Implementation

### Software overview

The *SIMAGE *web site [5] consists of three parts: (i) a start page where the user may upload parameters for a simulation; (ii) a web-page where the user specifies a number of parameters, after which (iii) the results, namely simulated data (see below), are presented. Each run is assigned a unique session, which allows the user to inspect the results at any given time. *SIMAGE *provides the user with three types of text files containing: (i) the parameters used for the simulation. This file can, in turn, be provided to *SIMAGE *for future simulations; (ii) the (differential) gene expressions, which are obtained after applying only the gene-layer; and (iii) the observed signals (including their location on the slide and the corresponding genes) after applying the layers explained in the following paragraphs. The estimates from a provided dataset are determined by a self-explanatory web site which is linked from the *SIMAGE *web site.

*SIMAGE *was written in Pascal and compiled by FreePascal version 1.0.10 [6]. Estimates for parameters, based on real data, are provided by a script developed in *R *[7]. The software requires an Apache web-server and is integrated with a PHP web-interface.

### The general model

Figure 1 shows a schematic representation of the model used in this study. Throughout this manuscript, and in the software package, the base-2 logarithm is used, unless stated otherwise. In this model the (log) expression signals are composed of the following components: (i) gene expression, (ii) a raw background gradient signal, (iii) a channel effect, (iv) a spot pin effect, (v) a nonlinear effect, (vi) a quantization and saturation effect, and (vii) random error due to unknown and/or unmodeled factors. The model is explained in more detail in the following text.

### Dimensions and notation

A DNA-microarray slide consists of a number of spots arranged in a two-dimensional matrix, which, in turn is divided into *n*_*row *_× *n*_*col *_square (sub)grids (see supplementary Fig. S1). Each grid contains nspot2
 MathType@MTEF@5@5@+=feaafiart1ev1aaatCvAUfKttLearuWrP9MDH5MBPbIqV92AaeXatLxBI9gBaebbnrfifHhDYfgasaacH8akY=wiFfYdH8Gipec8Eeeu0xXdbba9frFj0=OqFfea0dXdd9vqai=hGuQ8kuc9pgc9s8qqaq=dirpe0xb9q8qiLsFr0=vr0=vr0dc8meaabaqaciaacaGaaeqabaqabeGadaaakeaacqWGUbGBdaqhaaWcbaGaem4CamNaemiCaaNaem4Ba8MaemiDaqhabaGaeGOmaidaaaaa@34E0@ spots (*n*_*spot *_in both the horizontal and vertical direction), amounting to a total number of *n*_*row *_× *n*_*col *_× nspot2
 MathType@MTEF@5@5@+=feaafiart1ev1aaatCvAUfKttLearuWrP9MDH5MBPbIqV92AaeXatLxBI9gBaebbnrfifHhDYfgasaacH8akY=wiFfYdH8Gipec8Eeeu0xXdbba9frFj0=OqFfea0dXdd9vqai=hGuQ8kuc9pgc9s8qqaq=dirpe0xb9q8qiLsFr0=vr0=vr0dc8meaabaqaciaacaGaaeqabaqabeGadaaakeaacqWGUbGBdaqhaaWcbaGaem4CamNaemiCaaNaem4Ba8MaemiDaqhabaGaeGOmaidaaaaa@34E0@ spots per slide. In total, *n*_*slide *_slides are simulated with each spot providing a measure of the green (Cy3) and the red (Cy5) signal. The simulated log expression at spot (*i, j*) of array *l *and channel *k *is denoted by *y*_*ijkl*_.

### Gene expressions

To distinguish between non-regulated and regulated genes, the log ratio is considered. Assuming absence of other systematic deviations, the log ratio of the Cy3 and C5 channel signals log(Cy3/Cy5) = log(Cy3) - log(Cy5), is zero for non-regulated genes (with deviances only due to randomness), positive for up-regulated genes, and negative for down-regulated genes. The part of *y*_*jikl *_that is affected in this gene expression layer will be called yijkl*
 MathType@MTEF@5@5@+=feaafiart1ev1aaatCvAUfKttLearuWrP9MDH5MBPbIqV92AaeXatLxBI9gBaebbnrfifHhDYfgasaacH8akY=wiFfYdH8Gipec8Eeeu0xXdbba9frFj0=OqFfea0dXdd9vqai=hGuQ8kuc9pgc9s8qqaq=dirpe0xb9q8qiLsFr0=vr0=vr0dc8meaabaqaciaacaGaaeqabaqabeGadaaakeaacqWG5bqEdaqhaaWcbaGaemyAaKMaemOAaOMaem4AaSMaemiBaWgabaGaeiOkaOcaaaaa@34A8@. This yijkl*
 MathType@MTEF@5@5@+=feaafiart1ev1aaatCvAUfKttLearuWrP9MDH5MBPbIqV92AaeXatLxBI9gBaebbnrfifHhDYfgasaacH8akY=wiFfYdH8Gipec8Eeeu0xXdbba9frFj0=OqFfea0dXdd9vqai=hGuQ8kuc9pgc9s8qqaq=dirpe0xb9q8qiLsFr0=vr0=vr0dc8meaabaqaciaacaGaaeqabaqabeGadaaakeaacqWG5bqEdaqhaaWcbaGaemyAaKMaemOAaOMaem4AaSMaemiBaWgabaGaeiOkaOcaaaaa@34A8@ will be the same on all spots where the same gene *g *is spotted (see also formula 3), hence we can use the notation yg*
 MathType@MTEF@5@5@+=feaafiart1ev1aaatCvAUfKttLearuWrP9MDH5MBPbIqV92AaeXatLxBI9gBaebbnrfifHhDYfgasaacH8akY=wiFfYdH8Gipec8Eeeu0xXdbba9frFj0=OqFfea0dXdd9vqai=hGuQ8kuc9pgc9s8qqaq=dirpe0xb9q8qiLsFr0=vr0=vr0dc8meaabaqaciaacaGaaeqabaqabeGadaaakeaacqWG5bqEdaqhaaWcbaGaem4zaCgabaGaeiOkaOcaaaaa@3087@. We split yg*
 MathType@MTEF@5@5@+=feaafiart1ev1aaatCvAUfKttLearuWrP9MDH5MBPbIqV92AaeXatLxBI9gBaebbnrfifHhDYfgasaacH8akY=wiFfYdH8Gipec8Eeeu0xXdbba9frFj0=OqFfea0dXdd9vqai=hGuQ8kuc9pgc9s8qqaq=dirpe0xb9q8qiLsFr0=vr0=vr0dc8meaabaqaciaacaGaaeqabaqabeGadaaakeaacqWG5bqEdaqhaaWcbaGaem4zaCgabaGaeiOkaOcaaaaa@3087@ into two parts: *G*_*g*;*k*_, which is concerned with the 'true' expression of gene *g *on channel *k *before any other layer is applied, and *D*_*g*;*k*_, which is a (possible) deviation due to up- or down-regulation of gene *g*. The modeling of yg*
 MathType@MTEF@5@5@+=feaafiart1ev1aaatCvAUfKttLearuWrP9MDH5MBPbIqV92AaeXatLxBI9gBaebbnrfifHhDYfgasaacH8akY=wiFfYdH8Gipec8Eeeu0xXdbba9frFj0=OqFfea0dXdd9vqai=hGuQ8kuc9pgc9s8qqaq=dirpe0xb9q8qiLsFr0=vr0=vr0dc8meaabaqaciaacaGaaeqabaqabeGadaaakeaacqWG5bqEdaqhaaWcbaGaem4zaCgabaGaeiOkaOcaaaaa@3087@ is done via yg;k*
 MathType@MTEF@5@5@+=feaafiart1ev1aaatCvAUfKttLearuWrP9MDH5MBPbIqV92AaeXatLxBI9gBaebbnrfifHhDYfgasaacH8akY=wiFfYdH8Gipec8Eeeu0xXdbba9frFj0=OqFfea0dXdd9vqai=hGuQ8kuc9pgc9s8qqaq=dirpe0xb9q8qiLsFr0=vr0=vr0dc8meaabaqaciaacaGaaeqabaqabeGadaaakeaacqWG5bqEdaqhaaWcbaGaem4zaCMaei4oaSJaem4AaSgabaGaeiOkaOcaaaaa@32E4@ = *G*_*g*;*k *_+ *D*_*g*;*k*_. To start with *G*_*g*;*k*_, we assume that this latent (i.e. unobservable) variable is distributed as *G*_*g*;*k *_~ N(*μ*,σG2
 MathType@MTEF@5@5@+=feaafiart1ev1aaatCvAUfKttLearuWrP9MDH5MBPbIqV92AaeXatLxBI9gBaebbnrfifHhDYfgasaacH8akY=wiFfYdH8Gipec8Eeeu0xXdbba9frFj0=OqFfea0dXdd9vqai=hGuQ8kuc9pgc9s8qqaq=dirpe0xb9q8qiLsFr0=vr0=vr0dc8meaabaqaciaacaGaaeqabaqabeGadaaakeaaiiGacqWFdpWCdaqhaaWcbaGaem4raCeabaGaeGOmaidaaaaa@30AC@). Here, *μ *is the average expression value, and σG2
 MathType@MTEF@5@5@+=feaafiart1ev1aaatCvAUfKttLearuWrP9MDH5MBPbIqV92AaeXatLxBI9gBaebbnrfifHhDYfgasaacH8akY=wiFfYdH8Gipec8Eeeu0xXdbba9frFj0=OqFfea0dXdd9vqai=hGuQ8kuc9pgc9s8qqaq=dirpe0xb9q8qiLsFr0=vr0=vr0dc8meaabaqaciaacaGaaeqabaqabeGadaaakeaaiiGacqWFdpWCdaqhaaWcbaGaem4raCeabaGaeGOmaidaaaaa@30AC@ can be interpreted as the variation in gene expression. Hence, the expressions of non-differentially expressed genes are distributed symmetrically around *μ*, according to a normal distribution. For a comment on the normality assumption of *G*_*g*;*k*_, see the end of this paragraph. In most DNA-microarray data *G*_*g*;1 _and *G*_*g*;2 _are not statistically independent [8], which can be observed by considering a plot of Cy3 versus Cy5 signals. Therefore the covariance cov(*G*_*g*;1_,*G*_*g*;2_) = *ρ*σG2
 MathType@MTEF@5@5@+=feaafiart1ev1aaatCvAUfKttLearuWrP9MDH5MBPbIqV92AaeXatLxBI9gBaebbnrfifHhDYfgasaacH8akY=wiFfYdH8Gipec8Eeeu0xXdbba9frFj0=OqFfea0dXdd9vqai=hGuQ8kuc9pgc9s8qqaq=dirpe0xb9q8qiLsFr0=vr0=vr0dc8meaabaqaciaacaGaaeqabaqabeGadaaakeaaiiGacqWFdpWCdaqhaaWcbaGaem4raCeabaGaeGOmaidaaaaa@30AC@ is introduced, where *ρ *is the correlation coefficient between the signals from the two channels.

For each gene we model the probability of being up-, down- and non-regulated *π*_+_, *π*_-_, and *π*_0_= 1 - *π*_+ _- *π*_-_, respectively. For up-regulated genes the log ratio is increased by 2*μ*_*D *_and for down-regulated genes the log ratio is decreased by 2*μ*_*D*_. The effect of regulation is modeled via *D*_*g*;*k*_:

(Dg;1Dg;2)=(k1k2)μD, where (k1,k2)={(−1,1)if gene g is downregulated;(0,0)if gene g is nonregulated;(1,−1)if gene g is upregulated.     (1)
 MathType@MTEF@5@5@+=feaafiart1ev1aaatCvAUfKttLearuWrP9MDH5MBPbIqV92AaeXatLxBI9gBaebbnrfifHhDYfgasaacH8akY=wiFfYdH8Gipec8Eeeu0xXdbba9frFj0=OqFfea0dXdd9vqai=hGuQ8kuc9pgc9s8qqaq=dirpe0xb9q8qiLsFr0=vr0=vr0dc8meaabaqaciaacaGaaeqabaqabeGadaaakeaadaqadaqaauaabeqaceaaaeaacqWGebardaWgaaWcbaGaem4zaCMaei4oaSJaeGymaedabeaaaOqaaiabdseaenaaBaaaleaacqWGNbWzcqGG7aWocqaIYaGmaeqaaaaaaOGaayjkaiaawMcaaiabg2da9maabmaabaqbaeqabiqaaaqaaiabdUgaRnaaBaaaleaacqaIXaqmaeqaaaGcbaGaem4AaS2aaSbaaSqaaiabikdaYaqabaaaaaGccaGLOaGaayzkaaacciGae8hVd02aaSbaaSqaaiabdseaebqabaGccqqGSaalcqqGGaaicqqG3bWDcqqGObaAcqqGLbqzcqqGYbGCcqqGLbqzcqqGGaaidaqadaqaaiabdUgaRnaaBaaaleaacqaIXaqmaeqaaOGaeiilaWIaem4AaS2aaSbaaSqaaiabikdaYaqabaaakiaawIcacaGLPaaacqGH9aqpdaGabaqaauaabaqadiaaaeaadaqadaqaaiabgkHiTiabigdaXiabcYcaSiabigdaXaGaayjkaiaawMcaaaqaaiabbMgaPjabbAgaMjabbccaGiabbEgaNjabbwgaLjabb6gaUjabbwgaLjabbccaGiabdEgaNjabbccaGiabbMgaPjabbohaZjabbccaGiabbsgaKjabb+gaVjabbEha3jabb6gaUjabbkhaYjabbwgaLjabbEgaNjabbwha1jabbYgaSjabbggaHjabbsha0jabbwgaLjabbsgaKjabbUda7aqaamaabmaabaGaeeimaaJaeeilaWIaeeimaadacaGLOaGaayzkaaaabaGaeeyAaKMaeeOzayMaeeiiaaIaee4zaCMaeeyzauMaeeOBa4MaeeyzauMaeeiiaaIaem4zaCMaeeiiaaIaeeyAaKMaee4CamNaeeiiaaIaeeOBa4Maee4Ba8MaeeOBa4MaeeOCaiNaeeyzauMaee4zaCMaeeyDauNaeeiBaWMaeeyyaeMaeeiDaqNaeeyzauMaeeizaqMaee4oaSdabaWaaeWaaeaacqqGXaqmcqqGSaalcqGHsislcqqGXaqmaiaawIcacaGLPaaaaeaacqqGPbqAcqqGMbGzcqqGGaaicqqGNbWzcqqGLbqzcqqGUbGBcqqGLbqzcqqGGaaicqWGNbWzcqqGGaaicqqGPbqAcqqGZbWCcqqGGaaicqqG1bqDcqqGWbaCcqqGYbGCcqqGLbqzcqqGNbWzcqqG1bqDcqqGSbaBcqqGHbqycqqG0baDcqqGLbqzcqqGKbazcqqGUaGlaaaacaGL7baacaWLjaGaaCzcamaabmaabaGaeGymaedacaGLOaGaayzkaaaaaa@C919@

Although *μ*_*D *_is a fixed number, because of the sum yg;k*
 MathType@MTEF@5@5@+=feaafiart1ev1aaatCvAUfKttLearuWrP9MDH5MBPbIqV92AaeXatLxBI9gBaebbnrfifHhDYfgasaacH8akY=wiFfYdH8Gipec8Eeeu0xXdbba9frFj0=OqFfea0dXdd9vqai=hGuQ8kuc9pgc9s8qqaq=dirpe0xb9q8qiLsFr0=vr0=vr0dc8meaabaqaciaacaGaaeqabaqabeGadaaakeaacqWG5bqEdaqhaaWcbaGaem4zaCMaei4oaSJaem4AaSgabaGaeiOkaOcaaaaa@32E4@ = *G*_*g*;*k *_+ *D*_*g*;*k *_that is 'measured' this layer behaves as if regulated genes get a random sized shift up or down. Note that *G*_*g*;*k*_, as well as almost all other stochastic variables are modeled in *SIMAGE *as outcomes of normal distributions. Although this brings about some oversimplification, we consider this not really an issue or, in the words of Wit and McClure (2004) [8], 'the misspecification made by using a normal approximation is typically negligible'. In addition, the superposition of the various normally distributed layers does not imply that the generated expressions levels themselves are normally distributed. Furthermore, modeling of yg*
 MathType@MTEF@5@5@+=feaafiart1ev1aaatCvAUfKttLearuWrP9MDH5MBPbIqV92AaeXatLxBI9gBaebbnrfifHhDYfgasaacH8akY=wiFfYdH8Gipec8Eeeu0xXdbba9frFj0=OqFfea0dXdd9vqai=hGuQ8kuc9pgc9s8qqaq=dirpe0xb9q8qiLsFr0=vr0=vr0dc8meaabaqaciaacaGaaeqabaqabeGadaaakeaacqWG5bqEdaqhaaWcbaGaem4zaCgabaGaeiOkaOcaaaaa@3087@ as a combination of three (normal) densities (non-, up- and down-regulated genes) enables estimating the model parameters (see "gene expressions" below). A more extensive modeling of the gene expressions, although biologically somewhat more correct, will result in a significantly more complex estimation of parameters compared to the parameters described in this study.

### Replication variation

Spotting (spot pin effects), hybridization (non-uniform distribution of the labeled probe over the slide surface), and quantization (due to slide scanner properties) introduce variation in *y*_*ijkl *_in addition to the 'natural biological variation'. This natural variation is modeled as follows. When a simulated gene *g *is spotted on spot *ijkl*, random errors *ε*_*ij*1*l *_and *ε*_*ij*2*l *_are drawn from *N*(0,σε2
 MathType@MTEF@5@5@+=feaafiart1ev1aaatCvAUfKttLearuWrP9MDH5MBPbIqV92AaeXatLxBI9gBaebbnrfifHhDYfgasaacH8akY=wiFfYdH8Gipec8Eeeu0xXdbba9frFj0=OqFfea0dXdd9vqai=hGuQ8kuc9pgc9s8qqaq=dirpe0xb9q8qiLsFr0=vr0=vr0dc8meaabaqaciaacaGaaeqabaqabeGadaaakeaaiiGacqWFdpWCdaqhaaWcbaGae8xTdugabaGaeGOmaidaaaaa@3137@) and are added to the two replicated measurements yij1l*
 MathType@MTEF@5@5@+=feaafiart1ev1aaatCvAUfKttLearuWrP9MDH5MBPbIqV92AaeXatLxBI9gBaebbnrfifHhDYfgasaacH8akY=wiFfYdH8Gipec8Eeeu0xXdbba9frFj0=OqFfea0dXdd9vqai=hGuQ8kuc9pgc9s8qqaq=dirpe0xb9q8qiLsFr0=vr0=vr0dc8meaabaqaciaacaGaaeqabaqabeGadaaakeaacqWG5bqEdaqhaaWcbaGaemyAaKMaemOAaOMaeGymaeJaemiBaWgabaGaeiOkaOcaaaaa@3439@ and yij2l*
 MathType@MTEF@5@5@+=feaafiart1ev1aaatCvAUfKttLearuWrP9MDH5MBPbIqV92AaeXatLxBI9gBaebbnrfifHhDYfgasaacH8akY=wiFfYdH8Gipec8Eeeu0xXdbba9frFj0=OqFfea0dXdd9vqai=hGuQ8kuc9pgc9s8qqaq=dirpe0xb9q8qiLsFr0=vr0=vr0dc8meaabaqaciaacaGaaeqabaqabeGadaaakeaacqWG5bqEdaqhaaWcbaGaemyAaKMaemOAaOMaeGOmaiJaemiBaWgabaGaeiOkaOcaaaaa@343B@. Each gene will be replicated *n*_*rep *_times.

### Background surface variation

The signal distribution over the surface of the DNA-microarray glass-slide is affected by various factors, e.g. slide surface chemistry and hybridization effects, leading to an irregular distribution of the labeled probe on the slide [8-10]. The most simplistic way to model such gradient signals is by using a tilted plane, such that the signals on one side of the slide are higher than on the other. A more sophisticated method is that of Balagurunathan and coworkers (2002) [24] where a quadratic function is implemented. Such a function, however, is limited in the sense that it has only one local extreme (and some extremes located on the slide edges).

We use a method that allows for a number of local extremes to exist. A number *n*_*bg *_of bivariate normal densities with random parameter settings identifying the location and size on the slide are computed and subsequently multiplied by a random amplitude *I *(between ±1). Each density is computed by:

fm(i,j)=Im2πσbgx2σbgy21−ρbg2e[−12(1−ρbg2)((i−μbg,x)2σbg,x2−2ρ(i−μbg,x)(j−μbg,y)σbg,xσbg,y+(j−μbg,y)2σbg,y2)],     (2)
 MathType@MTEF@5@5@+=feaafiart1ev1aaatCvAUfKttLearuWrP9MDH5MBPbIqV92AaeXatLxBI9gBaebbnrfifHhDYfgasaacH8akY=wiFfYdH8Gipec8Eeeu0xXdbba9frFj0=OqFfea0dXdd9vqai=hGuQ8kuc9pgc9s8qqaq=dirpe0xb9q8qiLsFr0=vr0=vr0dc8meaabaqaciaacaGaaeqabaqabeGadaaakeaacqWGMbGzdaWgaaWcbaGaemyBa0gabeaakmaabmaabaGaemyAaKMaeiilaWIaemOAaOgacaGLOaGaayzkaaGaeyypa0ZaaSaaaeaacqWGjbqsdaWgaaWcbaGaemyBa0gabeaaaOqaaiabikdaYGGaciab=b8aWjab=n8aZnaaDaaaleaacqWGIbGycqWGNbWzcqWG4baEaeaacqaIYaGmaaGccqWFdpWCdaqhaaWcbaGaemOyaiMaem4zaCMaemyEaKhabaGaeGOmaidaaOWaaOaaaeaacqaIXaqmcqGHsislcqWFbpGCdaqhaaWcbaGaemOyaiMaem4zaCgabaGaeGOmaidaaaqabaaaaOGaemyzau2aaWbaaSqabeaadaWadaqaaiabgkHiTmaalaaabaGaeGymaedabaGaeGOmaiZaaeWaaeaacqaIXaqmcqGHsislcqWFbpGCdaqhaaadbaGaemOyaiMaem4zaCgabaGaeGOmaidaaaWccaGLOaGaayzkaaaaamaabmaabaWaaSaaaeaadaqadaqaaiabdMgaPjabgkHiTiab=X7aTnaaBaaameaacqWGIbGycqWGNbWzcqGGSaalcqWG4baEaeqaaaWccaGLOaGaayzkaaWaaWbaaWqabeaacqaIYaGmaaaaleaacqWFdpWCdaqhaaadbaGaemOyaiMaem4zaCMaeiilaWIaemiEaGhabaGaeGOmaidaaaaaliabgkHiTiabikdaYiab=f8aYnaalaaabaWaaeWaaeaacqWGPbqAcqGHsislcqWF8oqBdaWgaaadbaGaemOyaiMaem4zaCMaeiilaWIaemiEaGhabeaaaSGaayjkaiaawMcaamaabmaabaGaemOAaOMaeyOeI0Iae8hVd02aaSbaaWqaaiabdkgaIjabdEgaNjabcYcaSiabdMha5bqabaaaliaawIcacaGLPaaaaeaacqWFdpWCdaWgaaadbaGaemOyaiMaem4zaCMaeiilaWIaemiEaGhabeaaliab=n8aZnaaBaaameaacqWGIbGycqWGNbWzcqGGSaalcqWG5bqEaeqaaaaaliabgUcaRmaalaaabaWaaeWaaeaacqWGQbGAcqGHsislcqWF8oqBdaWgaaadbaGaemOyaiMaem4zaCMaeiilaWIaemyEaKhabeaaaSGaayjkaiaawMcaamaaCaaameqabaGaeGOmaidaaaWcbaGae83Wdm3aa0baaWqaaiabdkgaIjabdEgaNjabcYcaSiabdMha5bqaaiabikdaYaaaaaaaliaawIcacaGLPaaaaiaawUfacaGLDbaaaaGccqGGSaalcaWLjaGaaCzcamaabmaabaGaeGOmaidacaGLOaGaayzkaaaaaa@B5C9@

where the parameters are provided by the user. These densities are summed, together with a linear gradient with a random tilt lower than *s*, to constitute the background surface signals (see Fig. 1B, top panel; see also the supplementary document). An additive modeling of the background effect is chosen over a multiplicative model. As to the reasons why, see the supplementary document.

### Channel deviations, gene-dye interactions and spot pin deviation

The rationale behind the modeling of these three layers is the same: for each of the layer values a deviation, drawn from a normal distribution, is added to (a subset of) the Cy3 / Cy5 signals.

In some actual DNA-microarray experiments, the average Cy3 signal is significantly different from the average Cy5 signal obtained for a spot; reasons are: gene-to-gene variation in dye incorporating efficiencies, global differences in Cy3 and Cy5 abundance in the DNA preparations, and bleaching of the dyes (Cy5 is inactivated faster than Cy3). This effect is incorporated in the model by a random deviation *C*_1 _for *N*(0,σchannel2
 MathType@MTEF@5@5@+=feaafiart1ev1aaatCvAUfKttLearuWrP9MDH5MBPbIqV92AaeXatLxBI9gBaebbnrfifHhDYfgasaacH8akY=wiFfYdH8Gipec8Eeeu0xXdbba9frFj0=OqFfea0dXdd9vqai=hGuQ8kuc9pgc9s8qqaq=dirpe0xb9q8qiLsFr0=vr0=vr0dc8meaabaqaciaacaGaaeqabaqabeGadaaakeaaiiGacqWFdpWCdaqhaaWcbaGaem4yamMaemiAaGMaemyyaeMaemOBa4MaemOBa4MaemyzauMaemiBaWgabaGaeGOmaidaaaaa@3906@) to all Cy3-measurements and another random deviation *C*_2 _from this distribution to all Cy5-measurements.

Differential incorporation of dyes in DNA occurs due to (i) the preference of reverse transcriptase for the physically smaller Cy3 label in case of direct labeling and (ii) differential hybridization because of the physically larger Cy5 label. The effects differ between genes as a consequence of DNA size and the distribution of the labeled nucleotides in the DNA molecule [11,12]. Gene-dye interactions are embedded in the model similarly to the channel effect: for all measurements belonging to gene *g *and dye *d *a random deviation *X*_*gk *_from *N*(0,σg×d2
 MathType@MTEF@5@5@+=feaafiart1ev1aaatCvAUfKttLearuWrP9MDH5MBPbIqV92AaeXatLxBI9gBaebbnrfifHhDYfgasaacH8akY=wiFfYdH8Gipec8Eeeu0xXdbba9frFj0=OqFfea0dXdd9vqai=hGuQ8kuc9pgc9s8qqaq=dirpe0xb9q8qiLsFr0=vr0=vr0dc8meaabaqaciaacaGaaeqabaqabeGadaaakeaaiiGacqWFdpWCdaqhaaWcbaGaem4zaCMaey41aqRaemizaqgabaGaeGOmaidaaaaa@3454@) is added to the signal.

Spot pins show systematic deviations in the amount of probe delivered to the glass surface. The signals measured of targets spotted with one spot pin might differ significantly from those spotted with another spot pin. The *n*_*pin *_drawings *S*_*pin*(*ij*) _from *N*(0,σpin2
 MathType@MTEF@5@5@+=feaafiart1ev1aaatCvAUfKttLearuWrP9MDH5MBPbIqV92AaeXatLxBI9gBaebbnrfifHhDYfgasaacH8akY=wiFfYdH8Gipec8Eeeu0xXdbba9frFj0=OqFfea0dXdd9vqai=hGuQ8kuc9pgc9s8qqaq=dirpe0xb9q8qiLsFr0=vr0=vr0dc8meaabaqaciaacaGaaeqabaqabeGadaaakeaaiiGacqWFdpWCdaqhaaWcbaGaemiCaaNaemyAaKMaemOBa4gabaGaeGOmaidaaaaa@33BE@) are used to model these effects. Spot pin effects are equal for both channels.

### Non-linearity

(*M*, *A*) plots [13] from experimentally obtained DNA-microarray data often look somewhat curved (e.g. "banana shaped") and / or tilted, generally with higher deviations for lower *A *values (see "quantization and saturation" below). The *SIMAGE *model allows for non-linearity using a transformation *f*_*nl *_based on two parameters: *α*_1_, specifying the maximum amount of curvature to be allowed, and *α*_2_, which specifies the maximum amount of linear tilt. Although the transformation is relatively simple, the resulting data mimics experimental data closely enough with respect to non-linearity. The exact definition of the modeling of this layer and some examples of the resulting curvature is presented in the supplementary document.

### Fishtails

A common artifact of DNA-microarray data is that genes with a lower expression tend to be noisier. This is apparent in the 'fishtail' appearance of (*M*, *A*) graphs. The main reason for this artifact is mainly that spots with a lower signal are quantified with larger errors than spots with higher signals [14]. Another reason could be the 'over-transformation' of data due to the log-transformation, and that a less influential power transformation would be more appropriate to obtain normally distributed gene expressions. It would, however, be extremely complicated to use another transformation, since the property of logarithms that it turns multiplicative effects into additive effects is used throughout the complete *SIMAGE *model. We have to content ourselves with this approximation.

The fishtailing behavior is incorporated in *SIMAGE *by a parameter *δ *that inflates the log ratio for spots with an average expression *A*_*ijl *_below *μ *by a factor (1+δσM−2(Aijl−μ)2)
 MathType@MTEF@5@5@+=feaafiart1ev1aaatCvAUfKttLearuWrP9MDH5MBPbIqV92AaeXatLxBI9gBaebbnrfifHhDYfgasaacH8akY=wiFfYdH8Gipec8Eeeu0xXdbba9frFj0=OqFfea0dXdd9vqai=hGuQ8kuc9pgc9s8qqaq=dirpe0xb9q8qiLsFr0=vr0=vr0dc8meaabaqaciaacaGaaeqabaqabeGadaaakeaadaqadaqaaiabigdaXiabgUcaRGGaciab=r7aKjab=n8aZnaaDaaaleaacqWGnbqtaeaacqGHsislcqaIYaGmaaGcdaqadaqaaiabdgeabnaaBaaaleaacqWGPbqAcqWGQbGAcqWGSbaBaeqaaOGaeyOeI0Iae8hVd0gacaGLOaGaayzkaaWaaWbaaSqabeaacqaIYaGmaaaakiaawIcacaGLPaaaaaa@4154@. A higher value of *δ *will result in a stronger effect and *δ *= 0 indicates no fishtail effect. The parameter σM2
 MathType@MTEF@5@5@+=feaafiart1ev1aaatCvAUfKttLearuWrP9MDH5MBPbIqV92AaeXatLxBI9gBaebbnrfifHhDYfgasaacH8akY=wiFfYdH8Gipec8Eeeu0xXdbba9frFj0=OqFfea0dXdd9vqai=hGuQ8kuc9pgc9s8qqaq=dirpe0xb9q8qiLsFr0=vr0=vr0dc8meaabaqaciaacaGaaeqabaqabeGadaaakeaaiiGacqWFdpWCdaqhaaWcbaGaemyta0eabaGaeGOmaidaaaaa@30B8@ will be introduced below ("gene expressions"). Our model mimics biological replicates by randomizing the effect of *δ *for non-regulated genes. For each slide these genes have a probability of 1/2 of being influenced by fishtailing. In this way, the expressions of non-regulated genes are seldom distributed in such a way that they appear differentially regulated in a simulated experiment with replicated slides.

### Quantization and saturation

Slide scanning introduces another non-linearity artifact. The scanning device uses 16 bits to describe signal strength, i.e. signals are always between log(2^0^) = 0 and log(2^16^) = 16. Furthermore, some DNA-microarray data scanners tend to over-measure low values, and under-measure high values [15]. When no over- or under-measurement occurs, the signal transformation *l*_0_(*y*) = min(max(0,y),16) on *y *will be used to incorporate the cutoff at log(2^0^) = 0 and log(2^16^) = 16. A maximal over- or under-measurement effect will result in the signal transformation l1(y)=16/(1+exp⁡(2−y4))
 MathType@MTEF@5@5@+=feaafiart1ev1aaatCvAUfKttLearuWrP9MDH5MBPbIqV92AaeXatLxBI9gBaebbnrfifHhDYfgasaacH8akY=wiFfYdH8Gipec8Eeeu0xXdbba9frFj0=OqFfea0dXdd9vqai=hGuQ8kuc9pgc9s8qqaq=dirpe0xb9q8qiLsFr0=vr0=vr0dc8meaabaqaciaacaGaaeqabaqabeGadaaakeaacqWGSbaBdaWgaaWcbaGaeGymaedabeaakmaabmaabaGaemyEaKhacaGLOaGaayzkaaGaeyypa0JaeGymaeJaeGOnayJaei4la8YaaeWaaeaacqaIXaqmcqGHRaWkcyGGLbqzcqGG4baEcqGGWbaCdaqadaqaaiabikdaYiabgkHiTmaaleaaleaacqWG5bqEaeaacqaI0aanaaaakiaawIcacaGLPaaaaiaawIcacaGLPaaaaaa@439B@. This transformation inflates low signals, deflates high signals, and leaves medium signals unchanged.

Via a parameter *w *(*w *between 0 and 1) the *SIMAGE *model is instructed on the severity of the bias. The transformation *g*_*nl *_used is a weighted average of *l*_0 _and *l*_1_, namely *g*_*nl *_= (1 - *w*)*l*_0_(*y*) + *wl*_1_(*y*). The supplementary document provides a visualization of the effect of different choices of *w*.

### Missing data

In actual DNA-microarray data measurements may be missing. Reasons are: slide surface imperfections, hybridization effects, no DNA delivered to the glass surface during the spotting process or presence of fibers or dust particles. Our simulation model implements three types of missing data (with signals set to 0): (i) line-segments mimic 'hairs', (ii) 'donuts' comprising multiple spots mimic dirty areas and (iii) missing spots. The implementation of the model allows specifying the number of occurrences of these types of missing data as well as their maximum size and shape.

### The *SIMAGE *model

In the *SIMAGE *model 29 parameters have to be specified (Table 2), of which 6 are known constants (e.g. the number of spots in a grid). The model, which results from the components described above, is defined as follows: the simulated log expression at spot (*i, j*) of array *l *and channel *k *is denoted by *y*_*ijkl *_as:

yijkl=gnl(tδ(fnl(bgijl+zijkl)))⋅mijl, withzijkl=Ggene(ijl);k+Dgene(ijl);k+Ck+Spin(ij)+Xgene(ijl);k+εijkl     (3)
 MathType@MTEF@5@5@+=feaafiart1ev1aaatCvAUfKttLearuWrP9MDH5MBPbIqV92AaeXatLxBI9gBaebbnrfifHhDYfgasaacH8akY=wiFfYdH8Gipec8Eeeu0xXdbba9frFj0=OqFfea0dXdd9vqai=hGuQ8kuc9pgc9s8qqaq=dirpe0xb9q8qiLsFr0=vr0=vr0dc8meaabaqaciaacaGaaeqabaqabeGadaaakqaabeqaaiabdMha5naaBaaaleaacqWGPbqAcqWGQbGAcqWGRbWAcqWGSbaBaeqaaOGaeyypa0Jaem4zaC2aaSbaaSqaaiabd6gaUjabdYgaSbqabaGcdaqadaqaaiabdsha0naaBaaaleaaiiGacqWF0oazaeqaaOWaaeWaaeaacqWGMbGzdaWgaaWcbaGaemOBa4MaemiBaWgabeaakmaabmaabaGaemOyaiMaem4zaC2aaSbaaSqaaiabdMgaPjabdQgaQjabdYgaSbqabaGccqGHRaWkcqWG6bGEdaWgaaWcbaGaemyAaKMaemOAaOMaem4AaSMaemiBaWgabeaaaOGaayjkaiaawMcaaaGaayjkaiaawMcaaaGaayjkaiaawMcaaiabgwSixlabd2gaTnaaBaaaleaacqWGPbqAcqWGQbGAcqWGSbaBaeqaaOGaeiilaWIaeeiiaaIaee4DaCNaeeyAaKMaeeiDaqNaeeiAaGgabaGaemOEaO3aaSbaaSqaaiabdMgaPjabdQgaQjabdUgaRjabdYgaSbqabaGccqGH9aqpcqWGhbWrdaWgaaWcbaGaem4zaCMaemyzauMaemOBa4Maemyzau2aaeWaaeaacqWGPbqAcqWGQbGAcqWGSbaBaiaawIcacaGLPaaacqGG7aWocqWGRbWAaeqaaOGaey4kaSIaemiraq0aaSbaaSqaaiabdEgaNjabdwgaLjabd6gaUjabdwgaLnaabmaabaGaemyAaKMaemOAaOMaemiBaWgacaGLOaGaayzkaaGaei4oaSJaem4AaSgabeaakiabgUcaRiabdoeadnaaBaaaleaacqWGRbWAaeqaaOGaey4kaSIaem4uam1aaSbaaSqaaiabdchaWjabdMgaPjabd6gaUnaabmaabaGaemyAaKMaemOAaOgacaGLOaGaayzkaaaabeaakiabgUcaRiabdIfaynaaBaaaleaacqWGNbWzcqWGLbqzcqWGUbGBcqWGLbqzdaqadaqaaiabdMgaPjabdQgaQjabdYgaSbGaayjkaiaawMcaaiabcUda7iabdUgaRbqabaGccqGHRaWkcqWF1oqzdaWgaaWcbaGaemyAaKMaemOAaOMaem4AaSMaemiBaWgabeaakiaaxMaacaWLjaWaaeWaaeaacqaIZaWmaiaawIcacaGLPaaaaaaa@B3F0@

where

*gene*(*ijl*) the gene spotted at location (*i*, *j*,*l*) (see "dimensions and notation" above);

*pin*(*ij*) the spot pin used to spot location (*i*, *j*);

*g*_*nl *_a transformation due to quantization and saturation;

*t*_*δ *_a transformation due to 'fishtailing';

*f*_*nl *_a transformation due to non-linearity in measurements;

*m*_*ijkl *_equals 0 if the spot at location (*i*, *j*,*l*) is 'missing', and 1 otherwise (see "missing data" above);

*bg*_*ijl *_the background gradient level (see "background variation" above);

*G*_*g*;*k *_the expression level of gene *g *in channel *k*;

*D*_*g*;*k *_the change in expression due to up-/down-regulation;

*C*_*k *_the channel effect;

*S*_*pin*(*ij*) _the spot pin effect;

*X*_*gene*(*ijl*);*k *_the gene × dye interaction;

*ε*_*ijkl *_the replication error.

### Datasets used and estimation of the parameters

In order to estimate the parameters for a number of slides hybridized at the Department of Molecular Genetics (MolGen), DNA-microarray data from 47 *Lactococcus lactis *IL1403 slides from MolGen [3,16-18] were used (supplementary Table T1). These datasets specify, for each spot: (i) the annotated gene names, (ii) the raw expression values for both channels, and (iii) an estimated background signal. Parameters were determined from the net intensity values of the slides (i.e. corrected for background signals).

Besides MolGen slides, several slides from the public domain were analyzed. Datasets GDS69, GDS100, and GDS273 were obtained from the Gene Expression Omnibus from NCBI [19]. The dataset MEXP-225 was obtained from EBI's ArrayExpress [20]. In the supplementary web site [5], the exact slides used for the parameter estimations are listed.

The *SIMAGE *web site provides a tool for the estimation steps described in the following sections. In order to obtain robust estimates for the slides, some slides yielding obviously outlying estimates, i.e. when σchannel2
 MathType@MTEF@5@5@+=feaafiart1ev1aaatCvAUfKttLearuWrP9MDH5MBPbIqV92AaeXatLxBI9gBaebbnrfifHhDYfgasaacH8akY=wiFfYdH8Gipec8Eeeu0xXdbba9frFj0=OqFfea0dXdd9vqai=hGuQ8kuc9pgc9s8qqaq=dirpe0xb9q8qiLsFr0=vr0=vr0dc8meaabaqaciaacaGaaeqabaqabeGadaaakeaaiiGacqWFdpWCdaqhaaWcbaGaem4yamMaemiAaGMaemyyaeMaemOBa4MaemOBa4MaemyzauMaemiBaWgabaGaeGOmaidaaaaa@3906@ was larger than *μ*, were discarded. Only those experiments were used for which at least 3 slides with acceptable estimates were obtained. The estimates of the MolGen "experiment" were determined by the median of the "experiment" slides, while the MolGen "validation" estimates were determined by the median of the estimates of the validation slide data [3] (supplementary Table T1). For each of the 5 experiments, DNA-microarray data obtained from the same slide batch were used to estimate the experiment-specific parameters (see Results). This was done to minimize slide batch-specific bias in parameter estimation. The median, rather than the mean, of the estimates for each parameter is used as input in *SIMAGE *simulation interface because of the nonsymmetrical distribution of the estimates.

### Background gradient and densities

Spot background signals for the MolGen slides were determined as described [3]. Estimates for the number of background densities to be estimated, as well as their average standard deviations, were obtained by visually inspecting a number of experimentally obtained background densities. Furthermore, estimates for the average horizontal and vertical linear tilt were obtained by fitting a regression plane through the background signal distribution.

### Non-linearity, quantization and saturation

A direct way to estimate the scanning device bias parameter *w *is not feasible since this parameter is confounded within the other variables in the *SIMAGE *model. One could assume absence of scanning device bias (*w *= 0). We used an alternative method to estimate this parameter (for more details see the supplementary document). The estimation of the non-linearity parameters *α*_1 _and *α*_2 _is done as follows: in the (*M*, *A*) plot for background-corrected data (hence, the net signals), a Lowess curve [21] is fitted. Then a second degree polynomial is fitted to this Lowess curve, and the parameters *α*_1 _and *α*_2 _are directly derived from the parameters of the polynomial (see the supplementary document for details).

### Spot pin effects, channel effects, fishtails and replication variation

After subtracting the estimates for the background signals and the non-linearity effects from *y*_*ijkl *_(the original signal) we obtain *z*_*ijkl*_, where *z*_*ijkl *_= *G*_*gene*(*ijl*);*k *_+ *D*_*gene*(*ijl*);*k *_+ *C*_*k *_+ *S*_*pin*(*ij*) _+ *X*_*gene*(*ijl*);*k *_+ *ε*_*ijkl*_. The estimation of the gene layer factors *G *and *D *is discussed in the following paragraph. For all other factors, the estimation of their parameters is a matter of calculating a variance components model.

The gene-dye interaction is strongest when direct labeling of RNA is applied. An analysis by ANOVA of indirectly-labeled RNA used in self hybridization slides [3] and dye-swapped replicated slides (results not shown) performed at MolGen showed that the gene-dye interaction effects did not differ significantly. Note that the factor *X*_*gene*(*ijl*);*k *_can only reliably be estimated in case dye-swaps are performed with the same RNA.

The fishtail parameter *δ *was estimated by fitting a quadratic curve through the data points (*A*_*ijl*_,*|M*_*ijl*_*|*) with *A*_*ijl *_<*μ *(for details see the supplementary document).

### Gene expressions

**Figure 2:**
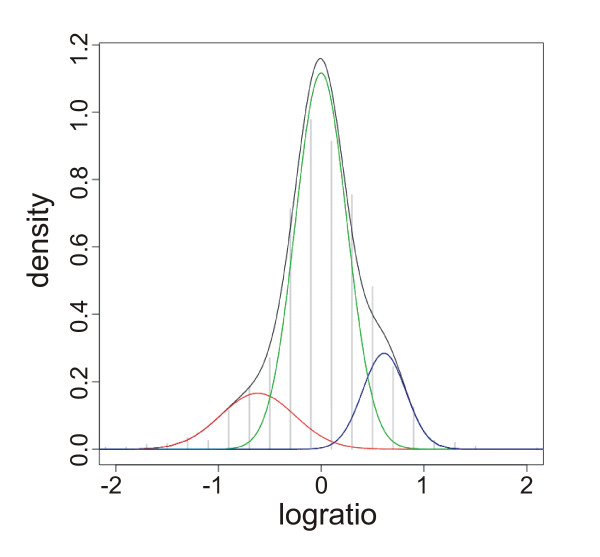
**The gene expression parameters**. These parameters (Table 2) were estimated by using the *EM*-algorithm (see "gene expressions" in the implementation section). The vertical lines constitute a stem-plot of the data. The red, green and blue curves indicate down-, not-, and up-regulated genes, respectively. The black curve is a combination of the three curves and, hence, the distribution of the logratios.

After correcting *z*_*ijkl *_for all factors except *G *and *D*, the parameters for *G *and *D *are estimated as follows: define Y˜g,k
 MathType@MTEF@5@5@+=feaafiart1ev1aaatCvAUfKttLearuWrP9MDH5MBPbIqV92AaeXatLxBI9gBaebbnrfifHhDYfgasaacH8akY=wiFfYdH8Gipec8Eeeu0xXdbba9frFj0=OqFfea0dXdd9vqai=hGuQ8kuc9pgc9s8qqaq=dirpe0xb9q8qiLsFr0=vr0=vr0dc8meaabaqaciaacaGaaeqabaqabeGadaaakeaacuWGzbqwgaacamaaBaaaleaacqWGNbWzcqGGSaalcqWGRbWAaeqaaaaa@31B8@ as the average value of the *n*_*rep*_replications *Y*_*ijkl *_with *gene*(*ijl*) = *g *: Y˜g;k=1nrep∑ijl|gene(ijl)=gYijl
 MathType@MTEF@5@5@+=feaafiart1ev1aaatCvAUfKttLearuWrP9MDH5MBPbIqV92AaeXatLxBI9gBaebbnrfifHhDYfgasaacH8akY=wiFfYdH8Gipec8Eeeu0xXdbba9frFj0=OqFfea0dXdd9vqai=hGuQ8kuc9pgc9s8qqaq=dirpe0xb9q8qiLsFr0=vr0=vr0dc8meaabaqaciaacaGaaeqabaqabeGadaaakeaacuWGzbqwgaacamaaBaaaleaacqWGNbWzcqGG7aWocqWGRbWAaeqaaOGaeyypa0ZaaSaaaeaacqaIXaqmaeaacqWGUbGBdaWgaaWcbaGaemOCaiNaemyzauMaemiCaahabeaaaaGcdaaeqbqaaiabdMfaznaaBaaaleaacqWGPbqAcqWGQbGAcqWGSbaBaeqaaaqaaiabdMgaPjabdQgaQjabdYgaSjabcYha8jabdEgaNjabdwgaLjabd6gaUjabdwgaLnaabmaabaGaemyAaKMaemOAaOMaemiBaWgacaGLOaGaayzkaaGaeyypa0Jaem4zaCgabeqdcqGHris5aaaa@543C@. Given the distributional assumptions outlined in "gene expressions" above, standard statistical theory provides that both the average log ratio (M˜g=Y˜g;1−Y˜g;2
 MathType@MTEF@5@5@+=feaafiart1ev1aaatCvAUfKttLearuWrP9MDH5MBPbIqV92AaeXatLxBI9gBaebbnrfifHhDYfgasaacH8akY=wiFfYdH8Gipec8Eeeu0xXdbba9frFj0=OqFfea0dXdd9vqai=hGuQ8kuc9pgc9s8qqaq=dirpe0xb9q8qiLsFr0=vr0=vr0dc8meaabaqaciaacaGaaeqabaqabeGadaaakeaacuWGnbqtgaacamaaBaaaleaacqWGNbWzaeqaaOGaeyypa0JafmywaKLbaGaadaWgaaWcbaGaem4zaCMaei4oaSJaeGymaedabeaakiabgkHiTiqbdMfazzaaiaWaaSbaaSqaaiabdEgaNjabcUda7iabikdaYaqabaaaaa@3AE0@) of the gene and the average intensity (A˜g=12(Y˜g;1+Y˜g;2)
 MathType@MTEF@5@5@+=feaafiart1ev1aaatCvAUfKttLearuWrP9MDH5MBPbIqV92AaeXatLxBI9gBaebbnrfifHhDYfgasaacH8akY=wiFfYdH8Gipec8Eeeu0xXdbba9frFj0=OqFfea0dXdd9vqai=hGuQ8kuc9pgc9s8qqaq=dirpe0xb9q8qiLsFr0=vr0=vr0dc8meaabaqaciaacaGaaeqabaqabeGadaaakeaacuWGbbqqgaacamaaBaaaleaacqWGNbWzaeqaaOGaeyypa0ZaaSqaaSqaaiabigdaXaqaaiabikdaYaaakmaabmaabaGafmywaKLbaGaadaWgaaWcbaGaem4zaCMaei4oaSJaeGymaedabeaakiabgUcaRiqbdMfazzaaiaWaaSbaaSqaaiabdEgaNjabcUda7iabikdaYaqabaaakiaawIcacaGLPaaaaaa@3E58@) of the gene are independently normally distributed, with variances σM2
 MathType@MTEF@5@5@+=feaafiart1ev1aaatCvAUfKttLearuWrP9MDH5MBPbIqV92AaeXatLxBI9gBaebbnrfifHhDYfgasaacH8akY=wiFfYdH8Gipec8Eeeu0xXdbba9frFj0=OqFfea0dXdd9vqai=hGuQ8kuc9pgc9s8qqaq=dirpe0xb9q8qiLsFr0=vr0=vr0dc8meaabaqaciaacaGaaeqabaqabeGadaaakeaaiiGacqWFdpWCdaqhaaWcbaGaemyta0eabaGaeGOmaidaaaaa@30B8@ and σA2
 MathType@MTEF@5@5@+=feaafiart1ev1aaatCvAUfKttLearuWrP9MDH5MBPbIqV92AaeXatLxBI9gBaebbnrfifHhDYfgasaacH8akY=wiFfYdH8Gipec8Eeeu0xXdbba9frFj0=OqFfea0dXdd9vqai=hGuQ8kuc9pgc9s8qqaq=dirpe0xb9q8qiLsFr0=vr0=vr0dc8meaabaqaciaacaGaaeqabaqabeGadaaakeaaiiGacqWFdpWCdaqhaaWcbaGaemyqaeeabaGaeGOmaidaaaaa@30A0@, respectively. The distribution A˜g
 MathType@MTEF@5@5@+=feaafiart1ev1aaatCvAUfKttLearuWrP9MDH5MBPbIqV92AaeXatLxBI9gBaebbnrfifHhDYfgasaacH8akY=wiFfYdH8Gipec8Eeeu0xXdbba9frFj0=OqFfea0dXdd9vqai=hGuQ8kuc9pgc9s8qqaq=dirpe0xb9q8qiLsFr0=vr0=vr0dc8meaabaqaciaacaGaaeqabaqabeGadaaakeaacuWGbbqqgaacamaaBaaaleaacqWGNbWzaeqaaaaa@2F49@ does not depend on whether gene *g *is up-, down- or non-regulated, and the parameters *μ *and σA2
 MathType@MTEF@5@5@+=feaafiart1ev1aaatCvAUfKttLearuWrP9MDH5MBPbIqV92AaeXatLxBI9gBaebbnrfifHhDYfgasaacH8akY=wiFfYdH8Gipec8Eeeu0xXdbba9frFj0=OqFfea0dXdd9vqai=hGuQ8kuc9pgc9s8qqaq=dirpe0xb9q8qiLsFr0=vr0=vr0dc8meaabaqaciaacaGaaeqabaqabeGadaaakeaaiiGacqWFdpWCdaqhaaWcbaGaemyqaeeabaGaeGOmaidaaaaa@30A0@ involved, as well as the variance σε2
 MathType@MTEF@5@5@+=feaafiart1ev1aaatCvAUfKttLearuWrP9MDH5MBPbIqV92AaeXatLxBI9gBaebbnrfifHhDYfgasaacH8akY=wiFfYdH8Gipec8Eeeu0xXdbba9frFj0=OqFfea0dXdd9vqai=hGuQ8kuc9pgc9s8qqaq=dirpe0xb9q8qiLsFr0=vr0=vr0dc8meaabaqaciaacaGaaeqabaqabeGadaaakeaaiiGacqWFdpWCdaqhaaWcbaGae8xTdugabaGaeGOmaidaaaaa@3137@, are estimated in a straightforward way. The mean of the distribution of M˜g
 MathType@MTEF@5@5@+=feaafiart1ev1aaatCvAUfKttLearuWrP9MDH5MBPbIqV92AaeXatLxBI9gBaebbnrfifHhDYfgasaacH8akY=wiFfYdH8Gipec8Eeeu0xXdbba9frFj0=OqFfea0dXdd9vqai=hGuQ8kuc9pgc9s8qqaq=dirpe0xb9q8qiLsFr0=vr0=vr0dc8meaabaqaciaacaGaaeqabaqabeGadaaakeaacuWGnbqtgaacamaaBaaaleaacqWGNbWzaeqaaaaa@2F61@ depends on whether the gene is up-, down- or non-regulated. The parameters *π*_-_, *π*_+_, *μ*_*D *_and σM2
 MathType@MTEF@5@5@+=feaafiart1ev1aaatCvAUfKttLearuWrP9MDH5MBPbIqV92AaeXatLxBI9gBaebbnrfifHhDYfgasaacH8akY=wiFfYdH8Gipec8Eeeu0xXdbba9frFj0=OqFfea0dXdd9vqai=hGuQ8kuc9pgc9s8qqaq=dirpe0xb9q8qiLsFr0=vr0=vr0dc8meaabaqaciaacaGaaeqabaqabeGadaaakeaaiiGacqWFdpWCdaqhaaWcbaGaemyta0eabaGaeGOmaidaaaaa@30B8@ are estimated using the *EM*-algorithm [22] (Figure 2) with some restrictions (see the supplementary document). Note that for self-self hybridizations *π*_- _= *π*_+ _= 0 and the estimation of σM2
 MathType@MTEF@5@5@+=feaafiart1ev1aaatCvAUfKttLearuWrP9MDH5MBPbIqV92AaeXatLxBI9gBaebbnrfifHhDYfgasaacH8akY=wiFfYdH8Gipec8Eeeu0xXdbba9frFj0=OqFfea0dXdd9vqai=hGuQ8kuc9pgc9s8qqaq=dirpe0xb9q8qiLsFr0=vr0=vr0dc8meaabaqaciaacaGaaeqabaqabeGadaaakeaaiiGacqWFdpWCdaqhaaWcbaGaemyta0eabaGaeGOmaidaaaaa@30B8@ is straightforward (it is the 'ordinary' variance of the *M*-values). Furthermore, fishtailing does not affect the spots with above-average intensities (*A*_*ijl *_> *μ*), nor the *A*-values. For all spots with average intensities higher than the overall average intensity, the *A*- and *M*-values are used to estimate parameters.

The estimates for σG2
 MathType@MTEF@5@5@+=feaafiart1ev1aaatCvAUfKttLearuWrP9MDH5MBPbIqV92AaeXatLxBI9gBaebbnrfifHhDYfgasaacH8akY=wiFfYdH8Gipec8Eeeu0xXdbba9frFj0=OqFfea0dXdd9vqai=hGuQ8kuc9pgc9s8qqaq=dirpe0xb9q8qiLsFr0=vr0=vr0dc8meaabaqaciaacaGaaeqabaqabeGadaaakeaaiiGacqWFdpWCdaqhaaWcbaGaem4raCeabaGaeGOmaidaaaaa@30AC@ and *ρ *(see "gene expressions" above), required for the *SIMAGE *model, are calculated from the estimates for σA2
 MathType@MTEF@5@5@+=feaafiart1ev1aaatCvAUfKttLearuWrP9MDH5MBPbIqV92AaeXatLxBI9gBaebbnrfifHhDYfgasaacH8akY=wiFfYdH8Gipec8Eeeu0xXdbba9frFj0=OqFfea0dXdd9vqai=hGuQ8kuc9pgc9s8qqaq=dirpe0xb9q8qiLsFr0=vr0=vr0dc8meaabaqaciaacaGaaeqabaqabeGadaaakeaaiiGacqWFdpWCdaqhaaWcbaGaemyqaeeabaGaeGOmaidaaaaa@30A0@ and σM2
 MathType@MTEF@5@5@+=feaafiart1ev1aaatCvAUfKttLearuWrP9MDH5MBPbIqV92AaeXatLxBI9gBaebbnrfifHhDYfgasaacH8akY=wiFfYdH8Gipec8Eeeu0xXdbba9frFj0=OqFfea0dXdd9vqai=hGuQ8kuc9pgc9s8qqaq=dirpe0xb9q8qiLsFr0=vr0=vr0dc8meaabaqaciaacaGaaeqabaqabeGadaaakeaaiiGacqWFdpWCdaqhaaWcbaGaemyta0eabaGaeGOmaidaaaaa@30B8@.

## Results

To illustrate the use of *SIMAGE *in drawing meaningful conclusions about the design and analysis of DNA microarray experiments we show a number of examples based on DNA microarray data generated within the MolGen department. *SIMAGE *is used as follows: (i) define parameters based on real DNA microarray data that are laboratory / experiment-specific, (ii) these parameters are roughly estimated by our estimation procedure, (iii) using these estimated parameters DNA microarray data is simulated mimicking real DNA microarray data as close as possible.

### The parameter estimation

**Figure 3:**
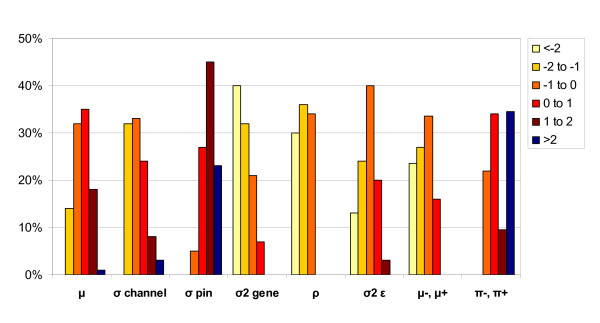
**Distribution of the deviations of several of the model parameters estimated from 100 simulated DNA-microarray slides. **The deviation is calculated as (estimate - true value) / (standard deviation of 100 estimates).

On the basis of a number of experiments that were performed at the MolGen department, the "MolGen experiment" parameters, 100 slides were simulated using *SIMAGE*. For each simulated slide, the model parameters were estimated using the estimation web-interface. Deviations of the mean and median of the estimated parameters from the original parameters are shown in Table 3 and Figure 3.

The estimation of *μ*, *σ*_channel _and *σ*_*ε *_are good (Table 3). Parameter *σ*_pin _is somewhat systematically overestimated (Fig. 3). The parameters σG2
 MathType@MTEF@5@5@+=feaafiart1ev1aaatCvAUfKttLearuWrP9MDH5MBPbIqV92AaeXatLxBI9gBaebbnrfifHhDYfgasaacH8akY=wiFfYdH8Gipec8Eeeu0xXdbba9frFj0=OqFfea0dXdd9vqai=hGuQ8kuc9pgc9s8qqaq=dirpe0xb9q8qiLsFr0=vr0=vr0dc8meaabaqaciaacaGaaeqabaqabeGadaaakeaaiiGacqWFdpWCdaqhaaWcbaGaem4raCeabaGaeGOmaidaaaaa@30AC@ and *ρ *tend to be systematically mildly underestimated. This is likely because of the nonsymmetric estimation of *ρ *(the true value of *ρ *is usually close to 1), which influences σG2
 MathType@MTEF@5@5@+=feaafiart1ev1aaatCvAUfKttLearuWrP9MDH5MBPbIqV92AaeXatLxBI9gBaebbnrfifHhDYfgasaacH8akY=wiFfYdH8Gipec8Eeeu0xXdbba9frFj0=OqFfea0dXdd9vqai=hGuQ8kuc9pgc9s8qqaq=dirpe0xb9q8qiLsFr0=vr0=vr0dc8meaabaqaciaacaGaaeqabaqabeGadaaakeaaiiGacqWFdpWCdaqhaaWcbaGaem4raCeabaGaeGOmaidaaaaa@30AC@ which is calculated from the estimate of *ρ*. The performance of the estimations of *δ*, *α*_1_, *α*_2 _and *w *depends highly on the actual values of these parameters. For low values of these parameters, they tend to be overestimated, while they are for high values underestimated. However, the estimation is rather good. As an indication, based on 100 slides with *δ*, *α*_1_, *α*_2_, and *w *all zero, the maxima of the 100 estimates for these parameters are 0.01, 0.004, 0.08, and 0.16 respectively.

The quality of the estimations of *μ*_-_, *μ*_+_, *π*_-_, *π*_+ _is highly experiment-dependent. Table 3 and Figure 3 show results for the choice of *μ*_- _= -1, *μ*_+ _= 1, *π*_- _= *π*_+ _= 10%, hence when the proportions of regulated genes and their average shifts in log ratio are considerable. The theory of Dempster and coworkers (1977) [22] indicates that the estimates of *μ*_-_, *μ*_+_, *π*_-_, *π*_+ _are unbiased: provided the correctness of our underlying statistical assumptions, no other estimators perform better. This is in concordance with the deviations listed in Table 3. In some cases where the true values of *μ*_-_, *μ*_+_, *π*_-_, and *π*_+ _are small their estimates are rather poor (Fig. 3). The user of the web-interface is suggested to 'tweak' these gene-expression estimates somewhat, if necessary.

### Within-laboratory experiment-dependency of estimated parameters

In order to investigate whether parameter estimates for different experiments from the same laboratory are significantly different, two profiles were generated: (i) "real" experiments [16-18] and (ii) validation experiments [3]. A Bonferonni-corrected Mann-Whitney test (*α *= 5%) showed that only parameters *μ *and *ρ *differ between the DNA-microarray data simulated using both profiles (supplementary Fig. S2, upper panel). Within the various "real" experiments, only the parameters describing regulation (*μ*_+_, *π*_+,_*μ*_-_, and *π*_-_) differ significantly (supplementary Fig. S2, lower panel). Parameters concerned with technical aspects of the DNA microarray spotter and scanner are, as expected, not significantly different, since the same equipment in one laboratory was used. Future studies on datasets obtained in other laboratories may implicate other parameters than those described above.

### Between-laboratory experiment-dependency of estimated parameters

**Figure 4:**
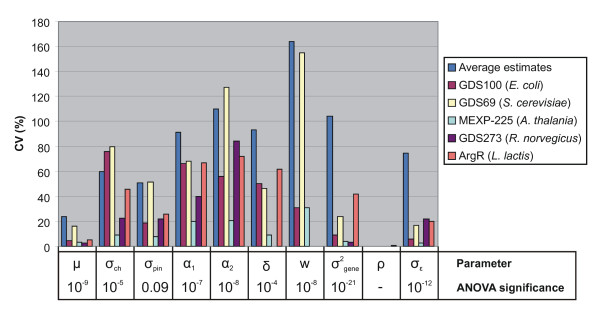
**Experiment-dependency of the parameters of the *SIMAGE *model. **The bar-graph shows the CVs ((standard deviation / average) × 100%) of the parameters, estimated from the individual datasets. The resulting CV was determined from the average estimates for each of the parameters obtained from the experiments. The *p*-value obtained by ANOVA is displayed below the parameter symbols; *p*-values below 0.05 are considered to be significant.

The model parameters were estimated from five different datasets, obtained by different laboratories and querying the mRNA levels of different organisms. The CVs of the model parameters obtained for individual datasets are, in general, lower than those obtained for the combined experiments (Fig. 4). Parameters which differ strongly between the datasets (combined CV values are higher than the CVs of the individual experiment) are: average expression (*μ*), tail behavior (*δ*), gene variance (*σ*^2^_gene_), and the general error (*σ*_*ε*_). For a few datasets the estimation of the parameters is quite "noisy", e.g. the CV for the non-linearity of scanner parameter (*w*) of the GDS69 dataset (Fig. 4). ANOVA shows that the estimated parameters, with the exception of the spot pin variation *σ*_pin _and the covariance *ρ*, are strongly experiment-dependent (Fig. 4). In particular the gene variance (*σ*^2^_gene_) is strongly influenced by the experiment (*p*-value of 10^-21^; Fig. 4).

### Graphical features of the simulated data

**Figure 5:**
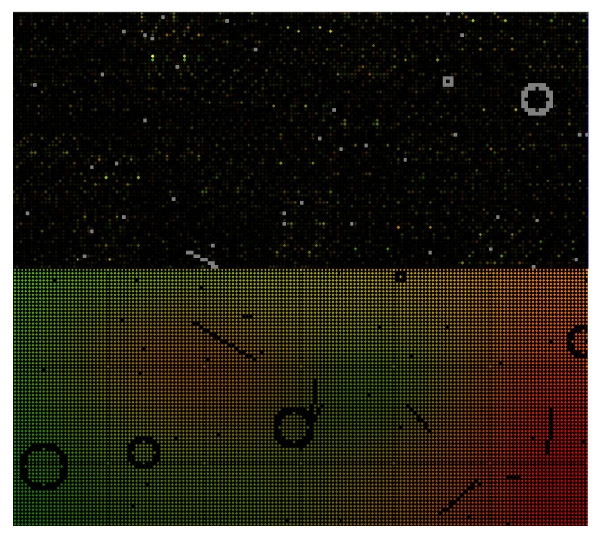
**Visualization of the signals of a simulated slide. **The upper picture shows a visualization of the measured expressions, while the lower picture is a visualization of the measured background signals. The areas designated as 'missing' are grey.

To ensure that the simulated data contains similar properties as experimentally obtained data, several aspects of the simulated slides were inspected. In supplementary Figure S3 three simulated and three "real" slides are visualized via an (*M*, *A*)-plot and by grid-based box plots. Several properties of the experimental slides are clearly present in a similar way in the simulated slides. Figure 5 shows a graphical representation of the net and background spot signals of a simulated slide.

### The modeled differentially expressed genes

**Figure 6:**
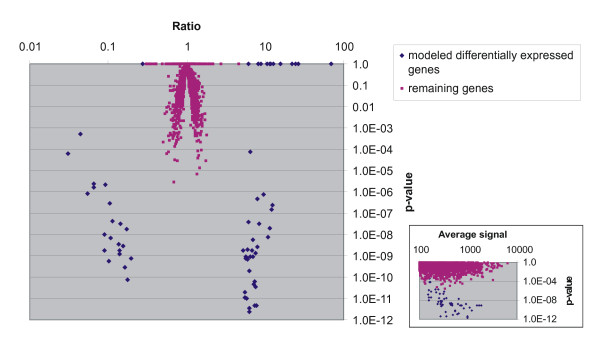
**Distribution of *p*-values of a DNA-microarray experiment simulated by *SIMAGE*. **Data for 2200 genes, in 6 slides with technical duplicates hybridized in dye-swaps, was simulated using the MolGen experiment profile (supplementary Table T1) with some changes: *π*_- _= 1% and *π*_+ _= 2%, *μ*_- _= -2 and *μ*_+ _= 2), *σ*_*bg *_= 700, and *s *= 30 % × *μ*. The main graph shows the resulting ratios after normalization plotted versus the *p*-value. The graph was simplified by removing genes with ratios between 2/3 and 3/2. The 66 genes for which differential expressions were modeled are depicted by blue diamonds. The remaining genes are depicted in purple squares. The small graph on the right demonstrates the reversed *p*-value dependency on the average signal for the 66 differentially expressed genes modeled. The average signal was calculated for each of the 66 genes over the maximum of 12 normalized measurements. Normalization was performed using Lowess normalization and differential expression tests were performed with the non-Bayesian Cyber-T implementation of a variant of the *t*-test [3]. The Cyber-T test provides the *p*-values, which indicate the probability that a given ratio is not differential caused by chance. Genes with less than 8 measurements were excluded from these tests and assigned a *p*-value of 1, in order to be able to present these genes in the graph.

*SIMAGE *allows modeling differentially expressed genes as is demonstrated in an *in silico *simulated experiment (Fig. 6). In almost all cases the *p*-values and ratios of the 66 modeled differentially expressed genes (the blue diamonds in Fig. 6) are significantly lower than those of the non-modeled genes (the red squares in Fig. 6). Relatively few of the known differentially expressed genes had signals that were close to the background signals: these genes get *p*-values close to 1. The inset in Figure 6 clearly demonstrates the signal dependency of the *p*-values: differentially expressed genes with higher expression levels are assigned lower *p*-values. This is in concordance with the p-value distributions in "real" DNA-microarray datasets.

## Discussion

As various factors, of both technical and biological nature, affect the quality of DNA-microarray data, it is essential to use a proper experiment design and sophisticated statistics and bioinformatics methods to deal with these variables. Since the factors involved, as well as their relative influence on data quality, vary between DNA-microarray laboratories and differ even between experiments executed in the same laboratory, the question as to which design and analysis method is best, cannot be answered in general terms. *SIMAGE*, a model and web-implementation to simulate gene expressions, requires the specification of up to 29 parameters. It allows simulating data that resemble experimental DNA-microarray data. To determine the relative contribution of the various parameters in DNA-microarray data is a knowledgeable task. A second web-implementation is provided to easily provide rough but reasonable estimates of most of these parameters from experimental DNA-microarray datasets. There is an important educational aspect about the simulation of DNA-microarray data, which is clearly illustrated in Figure 1B: it provides clear insights in the contribution of each background layer of the model to the measured signal.

Almost all parameters in the model are strongly dependent on the experiment performed (Fig. 4 and supplementary Fig. S2). This holds both for biological parameters in several different experiments from the same laboratory and for technical and biological parameters in experiments from different laboratories. The experiment-dependency of the *μ *estimations (Fig. 4) is obvious from the fact that the average signals in the prokaryote datasets are higher than those in the eukaryote datasets. This is due to the fact that prokaryotes generally express a larger complement of their genes. The experiment-dependency of the gene variance (*σ*^2^_gene_) might also be attributed to differences in gene expression in the different organisms interrogated in the DNA-microarray experiments discussed above. The latter is also clearly reflected by the high significance of the *σ*^2^_gene _parameter obtained by ANOVA.

Any method for simulation of DNA microarray data can be questioned and criticized, mainly because there is neither established theory for the relation between expression (observed) and factors involved (some can be observed, others are hidden), nor for the statistical distribution of differential expression given by various causes across genes. Choices about the statistical aspects of the data need to be made when building a simulation model such as *SIMAGE*. Different choices would lead to (slightly) different models with other 'optimal parameter estimation methods'. We have compared the distribution of simulated data (under various circumstances) with that of experimentally obtained data and adapted our model to be able to mimic experimental data as much as possible.

Creating a *SIMAGE*-like model to simulate data from other than dual-dye DNA microarray platforms might be interesting for future work. Our approach, using layers to model the factors involved, could be universally applied to simulate such data. This is, however, quite a task, since each type of DNA microarray platform has its own specific properties. Affymetrix, for instance, employs single-dye chips. The model of the synthesis of oligonucleotides on the chip surface and the different signals obtained for multiple probes would have to be quite sophisticated. Another interesting approach would be to expand *SIMAGE *to incorporate gene-regulatory interactions or genes involved in documented pathways. The use of simulated gene-regulatory networks would provide a powerful tool to estimate the efficiency of network reconstruction algorithms.

## Conclusion

A number of models for DNA microarray data simulation have recently been developed (Table 1). The question how to simulate DNA microarray data in the best way is not easily and straight-forwardly answered. There are many considerations that pose a researcher wanting to simulate DNA microarray data for difficult choices. We have developed *SIMAGE*: software that simulates dual-dye DNA microarray data. The model that we employ, although more advanced than existing models, is still a simplification of reality. To ensure that the simulated DNA microarray data mimics real data as close as possible the model is "fitted" onto "real" DNA microarray data.

## Availability and requirements

**Project name**: *SIMAGE*

**Project home page**: 

**Operating system(s)**: Runs on any JavaScript enabled web-browser.

**Programming languages**: PHP, Pascal, R, and shell scripting.

**Other requirements**: Additional files and figures are contained in the supplementary web site which is accessible from the above-mentioned web site.

**License**: The web-resource is freely accessible. Details concerning the conditions for using *SIMAGE *are available from the above-mentioned web site.

## Authors' contributions

SvH and CA conceived the *SIMAGE *concept and methodology. SvH programmed *SIMAGE *including the web-interface and CA programmed the estimator script. CA and SvH drafted the manuscript and generated the figures and tables. RJ, JK and OK critically read and revised the final manuscript. All authors read and approved the final manuscript.

## Figures and Tables

**Table 1:** Comparison of the properties of different DNA-microarray simulation models described in literature. '+' and '-' indicate availability of the indicated feature in the specified model. Note that the modeling of features in the specific models is usually not the same.

	Method	*SIMAGE*, this study	Lalush *et al. *[23]	Balagurunathan *et al. *[24]	Lonnstedt *et al. *[25]	Wierling *et al. *[26]	GE2
model implementation	tabulated gene expression data	+	-	-	-	-	+
	modeling of gene networks	-	-	-	-	-	+
	TIFF output	-	+	+	+	+	-
	software available	+ [5]	+ ^1^	-	-	-	+ [27]
	adjustable model	+	+	+	+	-	+/-
model effects	background surface pattern	+	-	+	-	+	-
	spot pin / grid effects	+	+	+	-	-	-
	channel effects	+	-	+	-	-	-
	non-linearity effects	+	-	+	-	-	+
	missing data	+	-	+	-	-	-
	'fishtailing'	+	-	+	-	-	+
	spot shape / size	-	+	+	-	-	-

1) C++ code available from author.

**Table 2:** Overview of parameters in the *SIMAGE *model. Some parameters are known (such as number of spots per grid), others should be set by the user (the flag indicates when the parameter can also be estimated from the data). Details concerning these parameters are discussed in the implementation section.

Parameter	Description	Can be estimated
*n*_*row*_	Number of grids per row	
*n*_*col*_	Number of grids per column	
nspot2 MathType@MTEF@5@5@+=feaafiart1ev1aaatCvAUfKttLearuWrP9MDH5MBPbIqV92AaeXatLxBI9gBaebbnrfifHhDYfgasaacH8akY=wiFfYdH8Gipec8Eeeu0xXdbba9frFj0=OqFfea0dXdd9vqai=hGuQ8kuc9pgc9s8qqaq=dirpe0xb9q8qiLsFr0=vr0=vr0dc8meaabaqaciaacaGaaeqabaqabeGadaaakeaacqWGUbGBdaqhaaWcbaGaem4CamNaemiCaaNaem4Ba8MaemiDaqhabaGaeGOmaidaaaaa@34E0@	Number of spots per grid	
*n*_*slide*_	Number of slides	
*n*_*rep*_	Number of technical replications	
*n*_*pin*_	Number of spot pins	
*μ*	Mean expression signal	v
*μ*_-, _*μ*_+_	Logratio-shift due to down- and upregulation	v
*π*_-_, *π*_+_	Proportion of down-, up-regulated genes	v
σG2 MathType@MTEF@5@5@+=feaafiart1ev1aaatCvAUfKttLearuWrP9MDH5MBPbIqV92AaeXatLxBI9gBaebbnrfifHhDYfgasaacH8akY=wiFfYdH8Gipec8Eeeu0xXdbba9frFj0=OqFfea0dXdd9vqai=hGuQ8kuc9pgc9s8qqaq=dirpe0xb9q8qiLsFr0=vr0=vr0dc8meaabaqaciaacaGaaeqabaqabeGadaaakeaaiiGacqWFdpWCdaqhaaWcbaGaem4raCeabaGaeGOmaidaaaaa@30AC@	Variation in gene expression	v
*ρ*	Correlation between Cy3 and Cy5 expression	v
σε2 MathType@MTEF@5@5@+=feaafiart1ev1aaatCvAUfKttLearuWrP9MDH5MBPbIqV92AaeXatLxBI9gBaebbnrfifHhDYfgasaacH8akY=wiFfYdH8Gipec8Eeeu0xXdbba9frFj0=OqFfea0dXdd9vqai=hGuQ8kuc9pgc9s8qqaq=dirpe0xb9q8qiLsFr0=vr0=vr0dc8meaabaqaciaacaGaaeqabaqabeGadaaakeaaiiGacqWFdpWCdaqhaaWcbaGae8xTdugabaGaeGOmaidaaaaa@3137@	Replication variation	v
*n*_*bg*_	Number of background 'densities'	
*σ*_*bg*_	Mean standard deviation per background density	
*s*	Maximum slope of the linear tilt	
σchannel2 MathType@MTEF@5@5@+=feaafiart1ev1aaatCvAUfKttLearuWrP9MDH5MBPbIqV92AaeXatLxBI9gBaebbnrfifHhDYfgasaacH8akY=wiFfYdH8Gipec8Eeeu0xXdbba9frFj0=OqFfea0dXdd9vqai=hGuQ8kuc9pgc9s8qqaq=dirpe0xb9q8qiLsFr0=vr0=vr0dc8meaabaqaciaacaGaaeqabaqabeGadaaakeaaiiGacqWFdpWCdaqhaaWcbaGaem4yamMaemiAaGMaemyyaeMaemOBa4MaemOBa4MaemyzauMaemiBaWgabaGaeGOmaidaaaaa@3906@	Channel variation	v
σpin2 MathType@MTEF@5@5@+=feaafiart1ev1aaatCvAUfKttLearuWrP9MDH5MBPbIqV92AaeXatLxBI9gBaebbnrfifHhDYfgasaacH8akY=wiFfYdH8Gipec8Eeeu0xXdbba9frFj0=OqFfea0dXdd9vqai=hGuQ8kuc9pgc9s8qqaq=dirpe0xb9q8qiLsFr0=vr0=vr0dc8meaabaqaciaacaGaaeqabaqabeGadaaakeaaiiGacqWFdpWCdaqhaaWcbaGaemiCaaNaemyAaKMaemOBa4gabaGaeGOmaidaaaaa@33BE@	Spot pin variation	v
σX2 MathType@MTEF@5@5@+=feaafiart1ev1aaatCvAUfKttLearuWrP9MDH5MBPbIqV92AaeXatLxBI9gBaebbnrfifHhDYfgasaacH8akY=wiFfYdH8Gipec8Eeeu0xXdbba9frFj0=OqFfea0dXdd9vqai=hGuQ8kuc9pgc9s8qqaq=dirpe0xb9q8qiLsFr0=vr0=vr0dc8meaabaqaciaacaGaaeqabaqabeGadaaakeaaiiGacqWFdpWCdaqhaaWcbaGaemiwaGfabaGaeGOmaidaaaaa@30CE@	Gene × dye variation	
*α*_1_	Non-linearity parameter 'curvature'	v
*α*_2_	Non-linearity parameter 'tilt'	v
*δ*	Fishtailing parameter	v
*w*	Scanning device bias	v
*n*_*h*_, *n*_*d*_, *n*_*s*_	Maximum number of hairs, donuts and missing spots	
*l*_*h*_, *l*_*d*_	Maximum length of hairs and radius of donuts	

**Table 3:** Estimation of parameters from the simulation of 100 DNA-microarray slides. The mentioned deviations are the number of estimated standard-deviations that the estimated mean, respectively median, lie away from the true value of the parameter.

Parameter	Deviation (mean)	Deviation (median)
*μ*	0.1	0.1
*σ*_channel_	-0.3	-0.5
*σ*_pin_	1.5	1.5
*σ*^2^_gene_	-1.6	-1.6
*ρ*	-1.6	-1.6
*σ*_*ε*_	-0.7	-0.6
|*μ*_-_|, *μ*_+_	-1.1	-1.0
*π*_-_, *π*_+_	1.0	0.7

## References

[B1] Piper MDW, ran-Lapujade P, Bro C, Regenberg B, Knudsen S, Nielsen J, Pronk JT (2002). Reproducibility of oligonucleotide microarray transcriptome analyses - An interlaboratory comparison using chemostat cultures of Saccharomyces cerevisiae. J Biol Chem.

[B2] Chen JJ, Delongchamp RR, Tsai CA, Hsueh HM, Sistare F, Thompson KL, Desai VG, Fuscoe JG (2004). Analysis of variance components in gene expression data. Bioinformatics.

[B3] Van Hijum SAFT, De Jong A, Baerends RJ, Karsens HA, Kramer NE, Larsen R, Den Hengst CD, Albers CJ, Kok J, Kuipers OP (2005). A generally applicable validation scheme for the assessment of factors involved in reproducibility and quality of DNA-microarray data. BMC Genomics.

[B4] Kerr MK, Martin M, Churchill GA (2000). Analysis of variance for gene expression microarray data. J Comput Biol.

[B5] (2006). The SIMAGE web-site. http://bioinformatics.biol.rug.nl/websoftware/simage.

[B6] (2006). The FreePascal homepage. http://www.freepascal.org.

[B7] (2006). The R project. http://www.r-project.org.

[B8] Wit E, McClure J (2004). Statistics for Microarrays - Design, Analysis and Inference.

[B9] Efron B, Tibshirani R, Storey J, Tusher V (2001). Empirical Bayes analysis of a microarray experiment. J Am Stat Assoc.

[B10] Wolkenhouer O, Moeller-Levet C, Sanchez-Cabo F (2002). The curse of normalization. Comp Func Genomics.

[B11] Dombkowski AA, Thibodeau BJ, Starcevic SL, Novak RF (2004). Gene-specific dye bias in microarray reference designs. FEBS Lett.

[B12] Martin-Magniette ML, Aubert J, Cabannes E, Daudin JJ (2005). Evaluation of the gene-specific dye bias in cDNA microarray experiments. Bioinformatics.

[B13] Dudoit S, Yang YH, Luu P, Speed TP (2001). Normalization for cDNA microarray data. Proc SPIE.

[B14] Widrow B, Kollár I, Liu MC (1996). Statistical theory of quantization. IEEE Trans Instrum Meas.

[B15] García de la Nava J, Van Hijum SAFT, Trelles O (2004). Saturation and Quantization Reduction in Microarray Experiments using Two Scans at Different Sensitivities. Stat Appl Gen Mol Biol.

[B16] Larsen R (2005). Transcriptional regulation of central amino acid metabolism in Lactococcus lactis.

[B17] Kramer NE (2005). Nisin-resistance in Gram-positive bacteria.

[B18] Den Hengst CD, Van Hijum SAFT, Geurts JM, Nauta A, Kok J, Kuipers OP (2005). The Lactococcus lactis CodY regulon: identification of a conserved cis-regulatory element. J Biol Chem.

[B19] (2006). The gene expression omnibus (GEO) from NCBI. http://www.ncbi.nlm.nih.gov/geo.

[B20] (2006). EBI databases - ArrayExpress home. http://www.ebi.ac.uk/arrayexpress.

[B21] Yang YH, Dudoit S, Luu P, Lin DM, Peng V, Ngai J, Speed TP (2002). Normalization for cDNA microarray data: a robust composite method addressing single and multiple slide systematic variation. Nucleic Acids Res.

[B22] Dempster AP, Laird NM, Rubin DB (1977). Maximum Likelihood from Incomplete Data Via EM Algorithm. J R Stat Soc Ser B Methodol.

[B23] Lalush DS, Lin S and Johnson K (2003). Characterization, modeling, and simulation of mouse microarray data. Methods of Microarray Data Analysis III.

[B24] Balagurunathan Y, Dougherty ER, Chen YD, Bittner ML, Trent JM (2002). Simulation of cDNA microarrays via a parameterized random signal model. J Biomed Opt.

[B25] Lonnstedt I, Speed T (2002). Replicated microarray data. Stat Sin.

[B26] Wierling CK, Steinfath M, Elge T, Schulze-Kremer S, Aanstad P, Clark M, Lehrach H, Herwig R (2002). Simulation of DNA array hybridization experiments and evaluation of critical parameters during subsequent image and data analysis. BMC Bioinformatics.

[B27] (2006). Gene expression data simulator. http://bioinformatics.upmc.edu/GE2/index.html.

